# Accuracy of toric intraocular lens power calculation depending on different keratometry values using a novel network based software platform

**DOI:** 10.3389/fmed.2024.1363286

**Published:** 2024-04-11

**Authors:** Michaela Ramsauer, Nikolaus Luft, Efstathios Vounotrypidis, Siegfried G. Priglinger, Wolfgang J. Mayer

**Affiliations:** ^1^Eye Clinic and Polyclinic, LMU Munich University Hospital, Munich, Germany; ^2^Department of Ophthalmology, University of Ulm, Ulm, Germany

**Keywords:** toric IOL calculation, SS-OCT-assisted biometry, Scheimpflug imaging, TK value, standard K front, TCRP, EQ workplace (Zeiss FORUM^®^)

## Abstract

**Purpose:**

To compare different corneal keratometry readings (swept-source-OCT-assisted biometry and Scheimpflug imaging) with a novel software platform for calculation of toric intraocular lenses.

**Setting:**

Department of Ophthalmology, Ludwig-Maximilians-University, Munich, Germany.

**Design:**

Retrospective, non-randomized, clinical trial.

**Methods:**

Twenty-three eyes undergoing toric intraocular lens implantation were included. Inclusion criteria were preoperative regular corneal astigmatism of at least 1.00 D, no previous refractive surgery, no ocular surface diseases and no maculopathies. Lens exchange was performed with CALLISTO eye (Zeiss). For each patient, the expected postoperative residual refraction was calculated depending on three different corneal parameters of two different devices: standard K-front (K) and total keratometry (TK) obtained by a swept-source-OCT-assisted biometry system (IOL Master 700, Zeiss) as well as total corneal refractive power (TCRP) obtained by a Scheimpflug device (Pentacam AXL, Oculus). Barrett’s formula for toric intraocular lenses was used for all calculations within a novel software platform (EQ workplace, Zeiss FORUM^®^). Results were statistically compared with postoperative refraction calculated according to the Harris dioptric power matrix.

**Results:**

The standard K values (mean PE 0.02 D ± 0.45 D) and TK values (mean PE 0.09 D ± 0.43 D) of the IOL Master 700 reached similar results (*p* = 0.96). 78% of eyes in both K and TK groups achieved SE within ±0.5 D of attempted correction and all eyes (100%) were within ±1.0 D of attempted correction in both groups. By contrast, the prediction error in the IOL calculation using the TCRP of the Scheimpflug device was significantly greater (mean PE −0.56 D ± 0.49 D; *p* = 0.00 vs. standard K and *p* = 0.00 vs. TK) with adjusted refractive indices. Thirty-nine and Ninety-one percentage of eyes in the TCRP group achieved SE within ±0.5 D (*p* = 0.008 K vs. TCRP and *p* = 0.005 TK vs. TCRP) and ± 1.0 D (*p* = 0.14 vs. TCRP) of attempted correction, respectively.

**Conclusion:**

All three corneal parameters (standard K, TK, TCRP) performed well in calculating toric IOLs. The most accurate refractive outcomes in toric IOL implantation were achieved by IOL calculations based on swept-source-OCT-assisted biometry. The SS-OCT-based K-front and TK values achieve comparable results in the calculation of toric IOLs.

## Introduction

The use of toric intraocular lenses (IOL) revolutionized cataract surgery and broadened the range of indications for refractive lens exchange. More than 20% of patients undergoing cataract surgery show a corneal astigmatism of 1.50 diopters (D) or higher (1, 2). Astigmatism results in image distortion due to varying magnification in the two principal meridians (3). Not surprisingly, the implantation of toric intraocular lenses gained massive importance in the past years.

While monofocal toric intraocular lenses focus sharply at one distance, multifocal toric IOLs produce a sharp image on the retina from several distances. However, to enable neutralization of corneal astigmatism, a precise preoperative calculation of toric IOL is of particular importance. One of the best indicators of IOL calculation accuracy, both in normal eyes and after refractive surgery as well as after implantation of a toric IOL, is the prediction error (PE), i.e., the deviation of preoperatively predicted residual refraction to the postoperatively determined refraction. Depending on the formula used, several parameters are included in the calculation of toric IOLs. The most important parameters are the axial length, the corneal power, and the lens position. The axial length is fundamental for calculating the power of all toric and non-toric intraocular lenses (IOLs) and its variation can lead to relevant refractive errors (4). To neutralize astigmatism, not only the sphere but also the precise toricity of the IOL must be calculated, that is primarily determined from the keratometry values of the cornea. Different devices use different methods for biometry. Two technologies that are frequently used are the swept-source-OCT (SS-OCT) technology and the rotating Scheimpflug camera. One of the most used SS-OCT-based biometric devices is the IOL Master 700 (Carl Zeiss Meditec AG, Jena, Germany). While a predecessor version of the machine, the IOL Master 500, was based on partial coherence interferometry (PCI) and was only able to measure the anterior surface of the cornea, the novel IOL Master 700 also measures the posterior corneal curvature. With information on the anterior and posterior corneal curvature, the IOL Master 700 can further calculate the total refractive power of the cornea (total keratometry, TK).

Unlike the IOL Master, the Pentacam (Oculus, Wetzlar, Germany) measures the total corneal refractive power (TCRP) using a rotating Scheimpflug camera.

The refractive results of toric intraocular lens implantation have been steadily improved in recent years.

Approaches for integrating potential influences of posterior corneal astigmatism into toric IOL calculations can broadly be categorized into two groups (5): estimation methods (such as adjusting nomograms and utilizing formulas based on population averages of posterior corneal astigmatism magnitude and orientation) and individual measurement of the posterior cornea (5). In theory, obtaining a precise individual measurement of an eye should yield more accurate refractive predictions compared to applying population-based statistics to the same eye. This iterative process across multiple eyes would theoretically demonstrate superior outcomes for individually measured eyes due to variations in posterior corneal astigmatism (5). However, empirical evidence has not consistently supported this anticipated distinction. Studies have shown that estimation methods for evaluating total corneal astigmatism are comparable to individual measurements (5, 6). Toric IOL calculation techniques based on estimated total corneal power have demonstrated even better performance than those incorporating individually measured total corneal power (7), except after refractive corneal surgery (5, 8).

The aim of this study is to investigate the accuracy of the toric IOL power calculation as a function of different corneal parameters (standard K-front, TK and TCRP) using a SS-OCT-based biometer and a commonly employed Scheimpflug device within a novel software platform (EQ workplace, Zeiss FORUM^®^).

## Materials and methods

This retrospective controlled study included 23 eyes treated with an aspheric hydrophilic toric intraocular lens (Zeiss, Germany) with plate haptic design (AT TORBI 709 M/MP in 16 eyes and AT LISA tri toric 939 M/MP in 7 eyes). Eighteen out of 23 eyes received cataract surgery, and 5 eyes underwent a refractive lens exchange. Optimized lens constants were used based on IOL Con database^1^ (9). Exclusion criteria included preoperative corneal astigmatism of less than 1.00 diopter (D), prior refractive surgery, corneal pathologies, ocular surface diseases and maculopathies.

Preoperatively, the implanted toric intraocular lenses were calculated based on the standard K-front and axial length (AL) measurement of a SS-OCT-based optical biometer (IOL Master 700, Carl Zeiss Meditec AG). Phacoemulsification and toric IOL implantation were performed by one experienced surgeon using a 2.2-mm steep-axis clear corneal incision (CCI) and the CALLISTO digital Eye Tracking system (Carl Zeiss Meditec AG) to adjust toric lens axis during standardized minimally invasive cataract surgery and refractive lens exchange. All procedures and study examinations were performed at the Department of Ophthalmology, Ludwig-Maximilians-University, Munich.

Postoperatively, based on the actually implanted toric intraocular lens, the expected postoperative residual refraction was calculated a second and third time for each patient using the preoperative TK values of the SS-OCT-based optical biometer (IOL Master 700), and the preoperative TCRP 3 mm zone obtained by a Scheimpflug device (Pentacam system AXL, Oculus), respectively. The IOL calculation was carried out with the EQ Workplace platform (Carl Zeiss Meditec AG) for each of the corneal parameters (standard K-front, TK, TCRP 3 mm zone). A further IOL calculation was performed with the TCRP using the device-integrated IOL calculator software of Pentacam AXL. In the sequel, the latter group is referred to as TCRP/Pentacam. For all calculations, the same AL measured preoperatively with the IOL Master 700 was used together with the Barrett’s formula for toric intraocular lenses and the different refractive indices (1.332 for standard K-front and TK and 1.3375 for TCRP) were considered. The actual postoperative alignment of the IOL axis was recorded at the slit lamp 3 weeks postoperatively.

The IOL calculations with the different corneal parameters suggested different values for the recommended IOL axis adjustment. To calculate the prediction error in each of the three groups, the postoperative refraction for each of the recommended positions of the IOL axis was calculated postoperatively using the Harris dioptric power matrix (10, 11). The Harris dioptric power matrix represents a universally applicable method for calculating astigmatism and refraction using a matrix representation of dioptric power. For detailed explanations, including mathematical formulas and sample calculations, see Supplementary material and the references of Harris (11) and Felipe et al. (10). To calculate the postoperative refraction in this way, a new keratometry was performed at least 7 days after surgery and the lens position was measured with the Pentacam device as the distance between the anterior corneal surface and the principal plane of the IOL in the visual axis of the eye. The deviation of the obtained postoperative and preoperative residual refraction in the respective groups (standard K-front, TK, TCRP/EQ Workplace, TCRP/Pentacam) were statistically compared with each other. The prediction error (PE) was defined as the difference between the spherical equivalent (SE) of the calculated postoperative refraction and predicted SE before the surgery.

In addition to the comparison of the various corneal parameters in the calculation of toric IOLs, diverse quality criteria of the surgery itself were determined: the reduction of cylinder, the surgically induced astigmatism (SIA), the misalignment of the IOL axis. In addition, the three different corneal parameters were compared with respect to the magnitude and axis of astigmatism.

Statistical evaluation was done with R (open source statistical program) using One-way ANOVA followed by Tukey’s multiple comparison test. The analysis of percentage of eyes that achieved SE within ±0.5 D and ± 1.00 D of attempted correction in the different groups was performed with Cochrane-*Q* test followed by Pairwise Cochran’s *Q* test using Excel Version 16.83. A *p*-value less than 0.05 was considered statistically significant. A post-hoc sample size calculation was performed using G*power software (12). Based on the results of the PE for the four different groups (with regard to the applied keratometry) we calculated *post hoc* the effect size f, which was 0.57. Given an *α*-error of 0.05, an effect size of 0.57 and a power of 0.8, a sample size of 10 eyes per group was calculated. The achieved power with 23 eyes per group in our study was 0.99.

## Results

The study evaluated the data of 23 eyes from 17 patients that underwent phacoemulsification with implantation of an aspheric hydrophilic toric intraocular lens (Zeiss, Germany) with plate haptic design. A total of 17 eyes exhibited with-the-rule (WTR, 60–120°) astigmatism, 5 eyes against-the-rule (ATR, 0–30° or 150–180°) astigmatism and 1 eye oblique (30–60° or 120–150°) astigmatism. Eight patients were men and 9 women. The mean age was 60 ± 9.9 (SD) years (range 42–79 years).

### Prediction error

**Figure 1:**
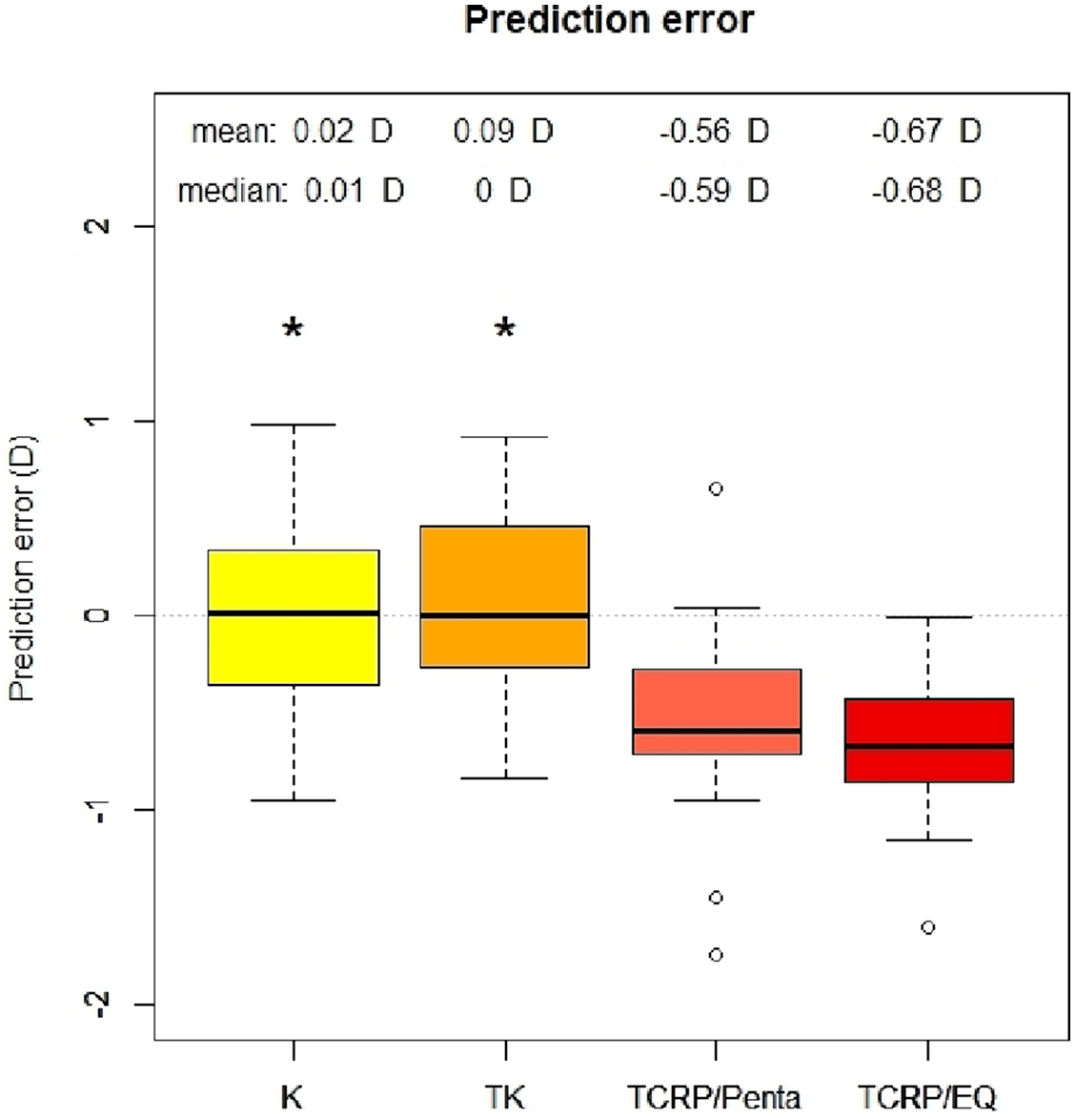
Prediction error of IOL calculation with standard keratometry (K), total keratometry (TK) and total corneal refractive power (TCRP). IOL calculation with TCRP was carried out once with Pentacam AXL (TCRP/Penta) and once with EQ Workplace (TCRP/EQ). (*n* = 23 each group; ^*^p < 0.05 vs. TCRP).

**Figure 2:**
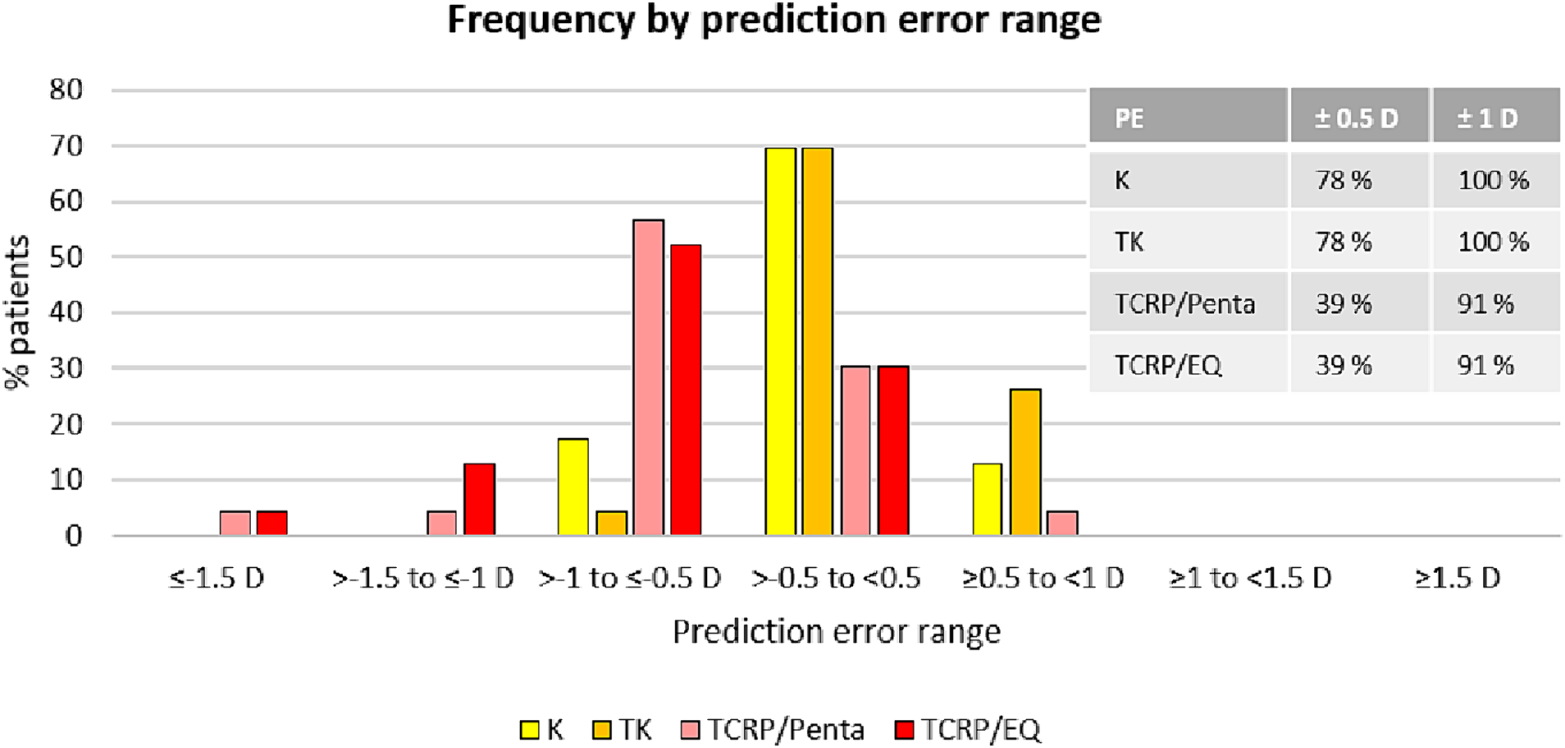
SE refraction accuracy: distribution of prediction errors in different groups [IOL calculation with K-values and TK-values using EQ Workplace, and with TCRP using the IOL calculator of the Pentacam AXL (TCRP/Penta) and using EQ Workplace (TCRP/EQ)].

The analysis of the prediction error (PE) in the four different groups (standard K, TK, TCRP/EQ Workplace, TCRP/Pentacam) is summarized in the Figures 1, 2. The lowest PE was found in the IOL calculations depending on standard K and TK values with a mean of 0.02 D ± 0.45 D and 0.09 D ± 0.43 D, respectively. There was no statistically significant difference in the PE between the standard K and the TK values (*p* = 0.96). In contrast, the prediction error was significantly greater depending on the TCRP, both calculating residual refraction with EQ Workplace (TCRP/EQ Workplace: mean PE −0.67 D ± 0.36 D; *p* = 0 vs. standard K and *p* = 0 vs. TK) and with the IOL calculator from Pentacam AXL (TCRP/Pentacam: mean PE −0.56 D ± 0.49 D; *p* = 0 vs. standard K and *p* = 0 vs. TK). There was no statistically significant difference in the PE between the two TCRP groups, TCRP/EQ Workplace and TCRP/Pentacam (*p* = 0.81). The percentage of eyes that achieved SE within ±0.5 D of attempted correction was 78% in both the K and TK groups, which was statistically significantly higher than in the TCRP group (39% of eyes achieving SE within ±0.5 D of attempted correction; *p* = 0.008 K vs. TCRP and *p* = 0.005 TK vs. TCRP). All eyes (100%) in the K and TK group achieved SE within ±1.0 D of attempted correction with no significant difference to the TCRP group (91% of eyes achieving SE within ±1 D of attempted correction; *p* = 0.14).

### Reduction of cylinder

**Figure 3:**
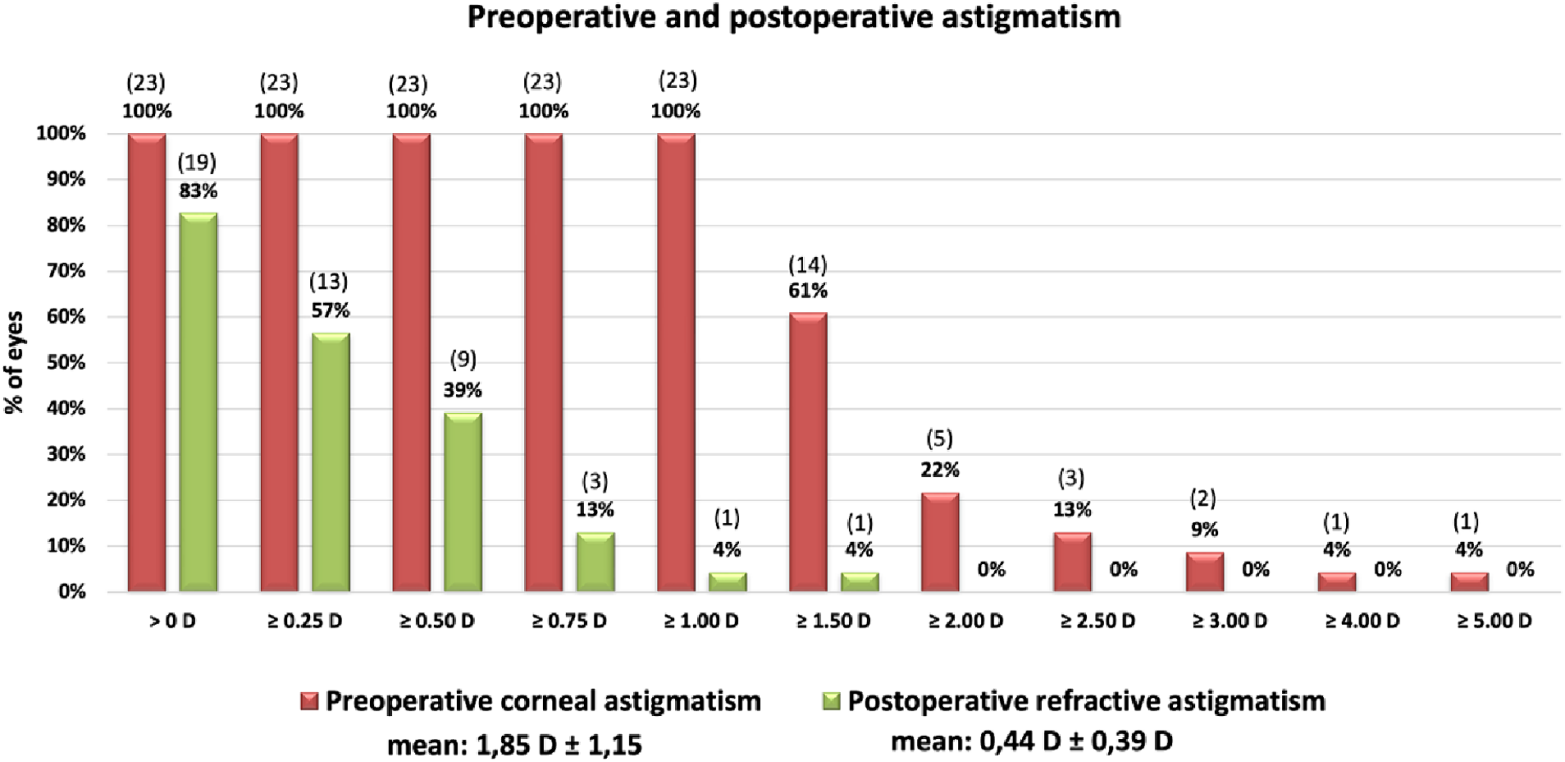
Cumulative histogram of the magnitudes of the preoperative corneal astigmatism (TCRP 3 mm) and the postoperative refractive cylinder at the corneal plane. Absolute number of patients in brackets.

**Figure 4:**
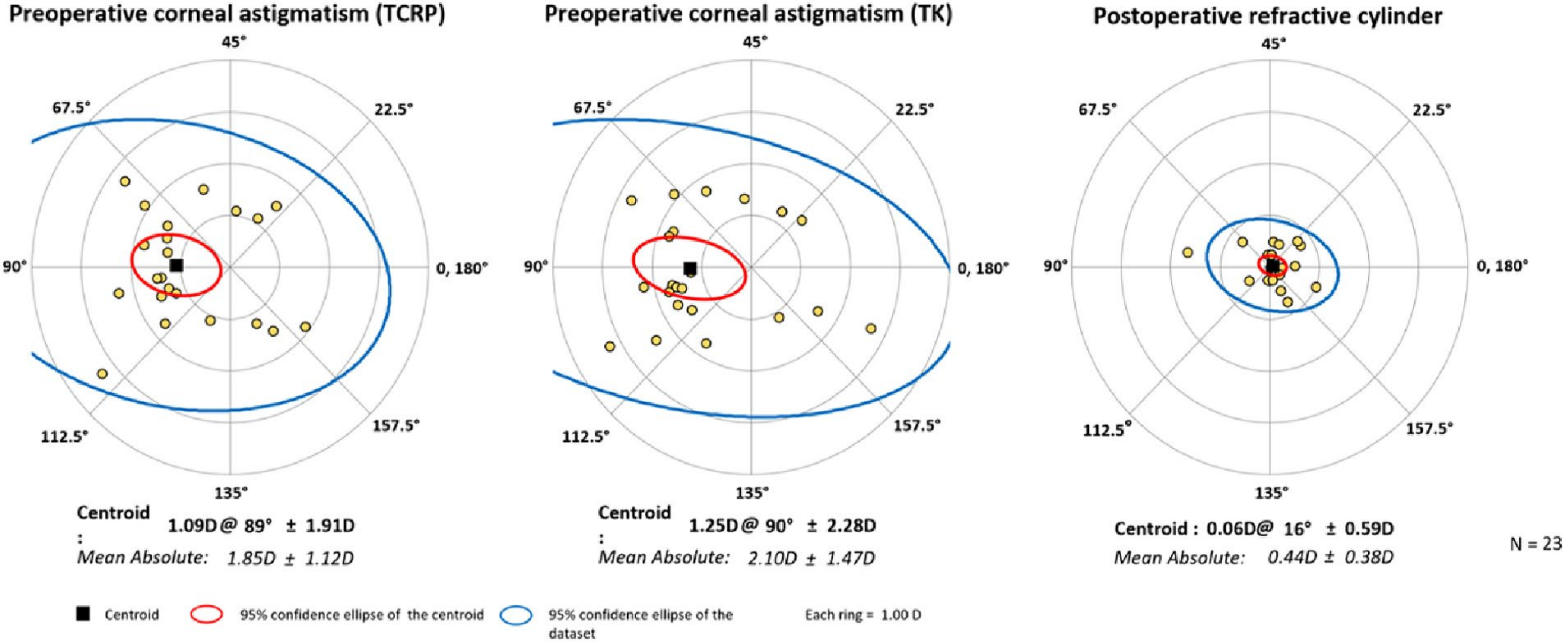
Double-angle plots of preoperative corneal astigmatism of TCRP (3 mm zone) and TK and postoperative refractive cylinder. [Created with Astigmatism Double Angle Plot Tool available on the ASCRS website (13) and described in Abulafia et al. (14).]

The surgery significantly reduced the mean cylinder of all patients from 1.85 D ± 1.15 D (TCRP) to 0.44 D ± 0.39 D (Figure 3). Vector analysis showed a reduction of preoperative Centroid of 1.09 D @ 89° ± 1.91 D (TCRP 3 mm) and 1.25 D @ 90° ± 1.91 D (TK), respectively, to postoperatively 0.06 D @ 16° ± 0.59 D refractive cylinder (Figure 4). In 52% of the eyes, residual refractive cylinder was within 0.25 D; in 74% it was within 0.5 D and in 96% it was within 1.0 D.

### Surgically induced astigmatism

The mean vector, or centroid, of the actual postoperative SIA of the cornea (SIA_Cornea_) was 0.22 D @ 15°. A SIA_Cornea_ of 0.37 D @ 2° was assumed preoperatively. Consequently, it was slightly overestimated. The SIA prediction error was 0.19 D @ 12°.

### IOL axial displacement

The axial displacement of the toric IOL from the intended axis was minimal and averaged 2.52° (±1.86°). 83% of all IOLs were rotated less than 5° and 100% less than 10°. No post rotation of any IOL was performed for the study collective.

### Differences in magnitude and axis of the astigmatism of the different corneal parameters

**Figure 5:**
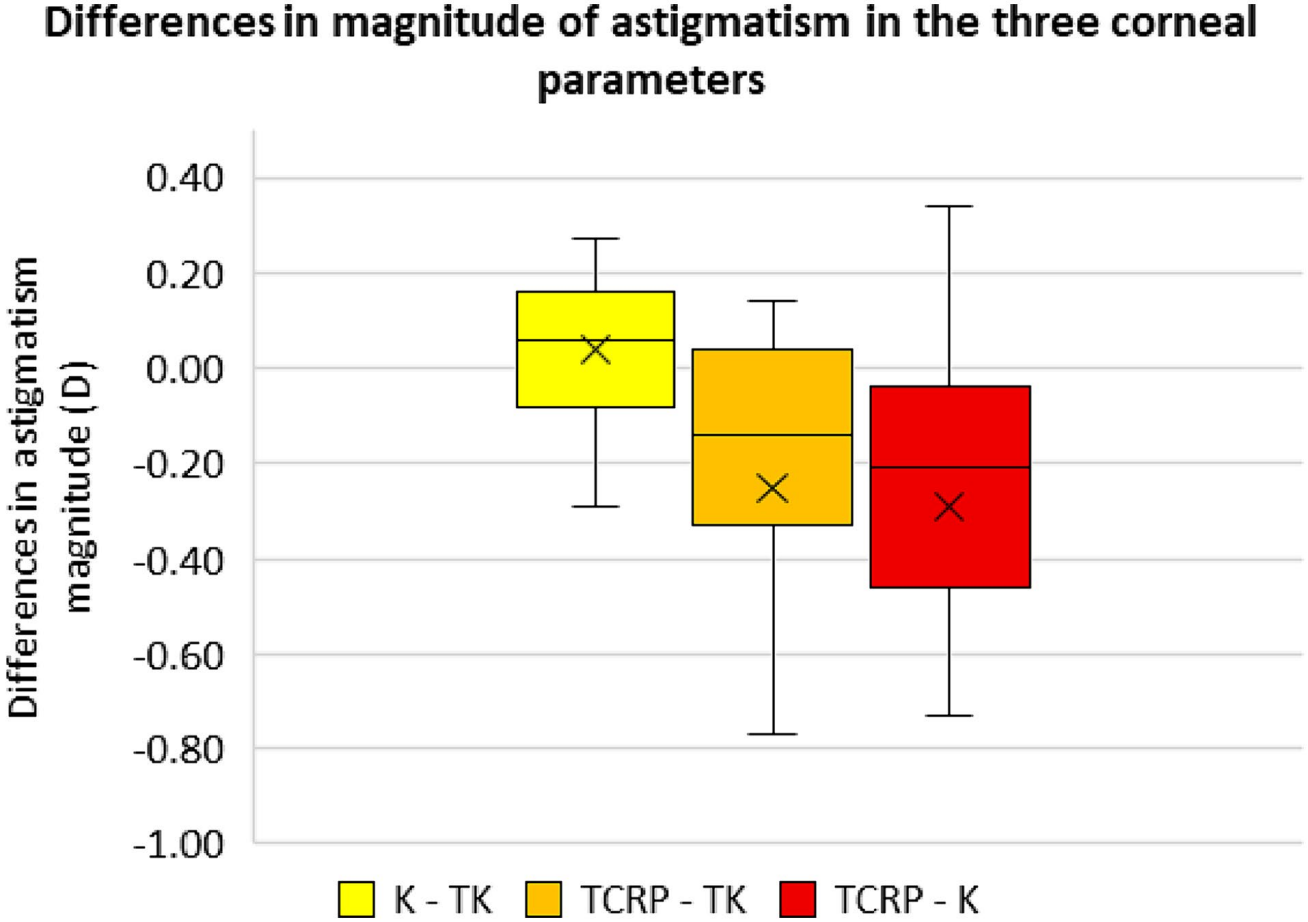
Differences in the magnitude of astigmatism in the three corneal parameters standard keratometry (K), total keratometry (TK) and total corneal refractive power (TCRP). (*n* = 23 each group).

**Figure 6:**
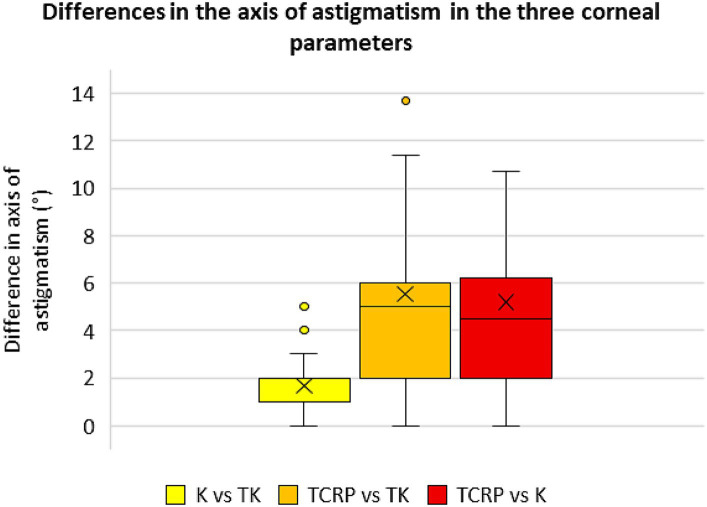
Differences in the axis of astigmatism in the three corneal parameters standard keratometry (K), total keratometry (TK) and total corneal refractive power (TCRP). (*n* = 23 each group).

Figures 5, 6 show the differences in astigmatism magnitude and axis of the three corneal parameters. Both magnitude and axis differed only slightly between the K and TK values, with a mean difference of 0.04 D ± 0.17 D in astigmatism magnitude and 1.6° ± 1.3° in axis. The differences between the TCRP and TK values were −0.25 D ± 0.47 D and 5.5° ± 5.4° for magnitude and axis, respectively, and between the TCRP and K values the differences were −0.29 D ± 0.50 D and 5.2° ± 4.8°, respectively.

## Discussion

The increasing use of toric intraocular lenses in cataract surgery and refractive lens exchange makes an exact calculation of these lenses indispensable. To date, there are few studies investigating the accuracy of toric IOL calculation depending on different corneal parameters or corneal parameters of different optical devices, respectively. Reitblat et al. (15) recommend the consideration of the posterior corneal astigmatism in the calculation of toric IOLs. The authors found that the residual astigmatism can be reduced by considering the back of the cornea in addition to the anterior surface. Conversely, ignoring the posterior corneal curvature may lead to misjudgment of the total corneal astigmatism resulting in significant postoperative over- or under-correction (15–18). However, a recently published study by Skrzypecki et al. (19) showed no statistically significant difference in astigmatism prediction errors with and without measured posterior corneal curvature, based on the IOL Master 700 biometry and the Barrett toric calculator.

The standard K values of the IOL Master 500 are well-proven in the calculation of standard lenses, but they only consider the anterior surface of the cornea. With the newer IOL Master 700 it is also possible to display the posterior corneal surface and the total refractive power.

In addition to PCI- and SS-OCT-based biometry, other optical biometers use Scheimpflug technology to measure the cornea. An example is the Pentacam, that also represents the total corneal refractive power (TCRP) by incorporating the anterior and posterior corneal surfaces. Davison et al. (20) showed that the TCRP achieves good results in toric IOL calculation. This is why some calculation formulas for toric IOLs are based on the TCRP, such as the Savini toric calculator (16). A direct comparison of the corneal parameters measured by SS-OCT and Scheimpflug technology in the calculation of toric IOLs, to the best of our knowledge, has not been analyzed so far.

In this study, we compared different corneal parameters with a novel software platform, the EQ Workplace, into which the data from the IOL Master 700 can be automatically transferred and different formulas and corneal radii can be used for IOL calculation, with further adjustment to different refractive indices. A subgroup analysis of the IOL calculation using the TCRP measured with the Scheimpflug device was performed with the device-integrated IOL calculator of the Pentacam AXL. We found that all three corneal parameters (standard K, TK, TCRP) performed well in calculating toric IOLs, with a mean prediction error of less than 0.7 D in all three groups. The standard K and TK values of the SS-OCT-based biometer provided such accurate results (mean PE of only 0.02 and 0.09 D, respectively) that they were statistically superior to the TCRP of the Scheimpflug device. In the K and TK group, 78% of the eyes were within 0.50 D of target refraction and all the eyes were within 1.0 D of target refraction. There was no statistically significant difference in the PE using the standard K and the TK values. In the TCRP groups (both TCRP/EQ and TCRP/Penta), 39% and 91% of the eyes were within 0.5 D and 1.0 D of target refraction, respectively.

As already mentioned, the different devices used in our study utilize different methods for measuring the anterior and posterior corneal curvature. The SS-OCT technology of the IOL Master 700 has a high scanning speed and delivers 2000 A-scans per second. One reason why the repeatability of the measurements is excellent. This was also shown by Srivannaboon et al. (21), who further demonstrated better lens penetration and axial length measurements compared to the previous model, the IOL Master 500. The Pentacam’s Scheimpflug camera rotates to produce Scheimpflug images from different perspectives. Because of the rotation of the Scheimpflug camera it takes a little more time, less than 2 s, to measure the anterior segment of the eye. Although reproducibility of the magnitude of the astigmatism with the Scheimpflug camera is good (22), McAlinden et al. (23) showed a lower reproducibility of the axis.

In this study, the magnitude and axis of astigmatism between the standard K and TK values differed only slightly, which also seems to be the reason why the standard K and TK values of the IOL Master 700 performed about equally well in terms of accuracy of toric IOL calculation. The differences between these SS-OCT-based corneal parameters and the TCRP of the Scheimpflug device were greater with respect to the magnitude and axis of astigmatism. In addition to our data, Hoffmann et al. (24) also showed that SS-OCT-based methods are best suited for calculating toric IOLs due to the precise corneal measurements. Nevertheless, Scheimpflug-based corneal examinations meanwhile become indispensable in clinical practice since unlike the IOL Master, it provides important information on the regularity of astigmatism over a larger tomographic map zone, especially when it comes to the indication of a toric IOL. When planning a toric IOL implantation, measurement of the cornea should be performed with at least two different methods and compared with each other. Identical results for value and axis of the astigmatism will increase their plausibility.

Our study has several limitations: The small number of patients may influence the validity of the study, so that the results need to be confirmed by a larger prospective controlled study. The inclusion of bilateral eyes cannot reliably exclude inter-eye correlations, that would affect the study results (25).

Our study included mostly patients with mild to moderate WTR astigmatism and without previous refractive surgery. Because of imprecise anterior keratometry measurements and the variation in keratometric index after refractive surgery, specific methods of IOL power calculation are required for these types of eyes (26). Some of these formulas also need the knowledge of anterior to posterior radii ratio (A/P ratio), that is altered after keratorefractive surgery (27).

Further studies are needed to investigate the accuracy of toric IOL calculation with different corneal parameters in eyes with higher astigmatism, larger differences between anterior and posterior corneal refractive power, e.g., in keratoconus patients, as well as in eyes with WTR, ATR, or oblique astigmatism only and after keratorefractive surgery. In addition, continuous improvements are making the devices more accurate, and there is an ever-increasing choice of formulas, including those with artificial intelligence, which offer more options for IOL calculation and comparison.

## Data availability statement

The raw data supporting the conclusions of this article will be made available by the authors, without undue reservation.

## Ethics statement

Ethical approval was not required for this retrospective study involving humans in accordance with the local legislation and institutional requirements. Written informed consent to participate in this study was not required from the participants or the participants’ legal guardians/next of kin in accordance with the national legislation and the institutional requirements.

## Author contributions

MR: Writing – review & editing, Writing – original draft, Validation, Software, Methodology, Investigation, Data curation, Conceptualization. NL: Visualization, Software, Resources, Writing – review & editing, Validation, Supervision, Project administration, Methodology, Formal analysis. EV: Writing – review & editing, Visualization, Validation, Supervision, Project administration, Methodology, Formal analysis, Conceptualization. SP: Writing – review & editing, Validation, Supervision, Software, Resources, Project administration. WM: Writing – review & editing, Visualization, Validation, Supervision, Software, Resources, Project administration, Methodology, Formal analysis, Data curation, Conceptualization.

## Footnotes

^1^ www.iolcon.org
