# Supplementary material for: Accuracy of toric intraocular lens power calculation depending on different keratometry values using a novel network based software platform
Source: Front Med (Lausanne). 2024 Apr 11;11:1363286. doi: 10.3389/fmed.2024.1363286 (PMC11043607; doi:10.3389/fmed.2024.1363286)
Supplement: Supplementary file 1 [file Data_Sheet_1.pdf]

Supplemental material:

Short explanation of Harris dioptric power matrix (for detailed explanations see references 8 and 9):

The IOL calculations with the different corneal parameters performed within our study suggested different values for the recommended IOL axis adjustment. To calculate the prediction error in each of the three groups, the postoperative refraction for each of the recommended positions of the IOL axis was calculated postoperatively using the Harris dioptric power matrix. The dioptric power matrix represents a universally applicable method for calculating astigmatism and refraction using a matrix representation of dioptric power. In accordance with this approach, the power matrix, denoted as F, of an astigmatic dioptric system is broken down into three orthogonal components:

$$F = F_{nes} + F_{ast} = F_{nes} + F_{or} + F_{ob} =$$

$$F_{nes}\begin{pmatrix} 1 & 0 \\ 0 & 1 \end{pmatrix} + F_{or}\begin{pmatrix} 1 & 0 \\ 0 & -1 \end{pmatrix} + F_{ob}\begin{pmatrix} 0 & 1 \\ 1 & 0 \end{pmatrix}$$

Here, F_{nes} (NES = nearest equivalent sphere) represents the purely spherical aspect of the overall power, while F_{ast} denotes the purely astigmatic aspect, further decomposing into orthoastigmatism (F_{or}) and oblique astigmatism (F_{ob}).

F is calculated both for the cornea (F_{Cornea}) and for the IOL (F_{IOL}) in the corneal plane. For this purpose, a new Pentacam image of the cornea of each eye was taken at least 7 days after the surgery. The IOL power in the corneal plane was calculated using precise determination of the postoperative lens position with the Pentacam device (measurement of the distance between the anterior corneal surface and the principal plane of the IOL in the visual axis of the eye). The total power of both the cornea and the IOL in the corneal plane is calculated as follows:

$$F_{total} = F_{Cornea} + F_{IOL} = F_{nes,total} + F_{or,total} + F_{ob,total}$$

with:

$$F_{nes,total} = F_{nes,Cornea} + F_{nes,IOL}$$

$$F_{or,total} = F_{or,Cornea} + F_{or,IOL}$$

$$F_{ob,total} = F_{ob,Cornea} + F_{ob,IOL}$$

To determine the refractive power of the spherocylindrical system of the eye (S C x α) in the corneal plane, where S is the sphere, C is the cylinder and α is the cylinder axis, the following formulas can be used:

$\tan(2\alpha) = F_{ob} / F_{or} \rightarrow \alpha = \arctan(F_{ob,total} / F_{or,total}) / 2$

$F_{or} = -C / 2 * \cos(2\alpha) \rightarrow C = F_{or,total} / \cos(2\alpha) * (-2)$

$F_{ob} = -C / 2 * \sin(2\alpha) \rightarrow C = F_{ob,total} / \sin(2\alpha) * (-2)$

$F_{nes} = S + C / 2 \rightarrow S = F_{nes,total} + (-C) / 2$

Subsequently, the optimal refractive power in the corneal plane for emmetropia was calculated as follows:

Optimal refractive power in the corneal plane = 1.336 / (AL / 1000)

The refractive error in the corneal plane is then calculated as follows:

Sphere (refractive error in the corneal plane) = ($F_{nes,total}$ - C/2) - optimal refractive power in the corneal plane

The cylinder in the corneal plane and the cylinder axis are calculated as described above:

$C_{corneal\ plane} = F_{or,total} * (-2) / \cos(2\alpha)$

$\alpha = \arctan(F_{ob,total} / F_{or,total}) / 2$
